# A BMPR2/YY1 Signaling Axis Is Required for Human Cytomegalovirus Latency in Undifferentiated Myeloid Cells

**DOI:** 10.1128/mBio.00227-21

**Published:** 2021-06-01

**Authors:** Emma Poole, Maria Cristina Carlan da Silva, Chris Huang, Marianne Perera, Sarah Jackson, Ian J. Groves, Mark Wills, Amer Rana, John Sinclair

**Affiliations:** a Cambridge Institute of Therapeutic Immunology and Infectious Disease, University of Cambridge School of Clinical Medicine, Cambridge Biomedical Campus, Cambridge, United Kingdom; b Department of Medicine, University of Cambridge School of Clinical Medicine, Cambridge Biomedical Campus, Cambridge, United Kingdom; c Center for Natural and Humanities Sciences, Federal University of ABC (UFABC), São Bernardo do Campo, Brazil; d Department of Medicine, University of Cambridge, Addenbrooke’s Hospital, Cambridge, United Kingdom; University of Wisconsin-Madison; University of North Carolina, Chapel Hill

**Keywords:** human cytomegalovirus, latency, BMPR2, YY1, stem cells

## Abstract

Human cytomegalovirus (HCMV) presents a major health burden in the immunocompromised and in stem cell transplant medicine. A lack of understanding about the mechanisms of HCMV latency in undifferentiated CD34^+^ stem cells, and how latency is broken for the virus to enter the lytic phase of its infective cycle, has hampered the development of essential therapeutics. Using a human induced pluripotent stem cell (iPSC) model of HCMV latency and patient-derived myeloid cell progenitors, we demonstrate that bone morphogenetic protein receptor type 2 (BMPR2) is necessary for HCMV latency. In addition, we define a crucial role for the transcription factor Yin Yang 1 (YY1) in HCMV latency; high levels of YY1 are maintained in latently infected cells as a result of BMPR2 signaling through the SMAD4/SMAD6 axis. Activation of SMAD4/6, through BMPR2, inhibits TGFbeta receptor signaling, which leads to the degradation of YY1 via induction of a cellular microRNA (miRNA), hsa-miR-29a. Pharmacological targeting of BMPR2 in progenitor cells results in the degradation of YY1 and an inability to maintain latency and renders cells susceptible to T cell killing. These data argue that BMPR2 plays a role in HCMV latency and is a new potential therapeutic target for maintaining or disrupting HCMV latency in myeloid progenitors.

## INTRODUCTION

Human cytomegalovirus (HCMV) is the prototypic betaherpesvirus, which, like all herpesviruses, establishes a lifelong infection in the host, with seroprevalence ranging from 60 to 90% depending on socioeconomic status. As such, HCMV presents a major health burden in the immunocompromised and in the stem cell as well as solid-organ-transplant setting. For example, HCMV seropositive stem cell or solid organ grafts pose a risk to transplant recipients where HCMV reactivation can cause significant posttransplant disease (1–3). HCMV is species specific, and therefore, there are no simple animal models of HCMV latency. However, *ex vivo* and *in vitro* analyses have allowed the HCMV life cycle to be studied in some detail. Full HCMV productive infection occurs in cells such as fibroblasts and epithelial as well as endothelial cells and comprises three phases. Initially, immediate early (IE) gene expression occurs, followed by early (E) gene expression and, finally, late gene expression, which ultimately leads to the production of infectious virions. Following primary infection in the host, latency can be established in, for instance, CD34^+^ myeloid progenitor cells or their derivative CD14^+^ monocytes, during which there is a general repression of IE gene expression and a lack of infectious virion production. One established model of HCMV latency is in the myeloid lineage, where undifferentiated myeloid cells support latency but, upon differentiation of these cells to terminally differentiated macrophages or dendritic cells (DCs), IE gene expression is initiated, leading to the full lytic life cycle and production of infectious virions (4–7).

Although the study of HCMV has been, at least partially, limited by the lack of an animal model, work in cell lines has been very informative. Undifferentiated myelomonocytic cell lines such as THP1 cells or Kasumi-3 cells as well as the undifferentiated teratocarcinoma cell line NT2D1 can establish latency-type infections, after which these cells can all be differentiated in culture to allow permissiveness for lytic infection. Such models have allowed aspects of HCMV latency and reactivation to be analyzed after infection with clinical isolates of HCMV, albeit with some of the limitations inherent with using nonprimary cell lines (8–13). Primary cells of the myeloid lineage, cultured *ex vivo*, have also been used extensively to develop experimental models of HCMV latency, but these also have the disadvantage of donor variability and a short life span. More recently, induced pluripotent stem cell (iPSC) models, which allow the culture of renewable populations of undifferentiated cells which can be differentiated along multiple lineages, have been used successfully to study HCMV latent infection and reactivation (14–16).

Consistently, the use of such models has shown that in undifferentiated cells nonpermissive for lytic infection, the chromatin structure around the major immediate early promoter (MIEP), which drives expression of the HCMV major lytic IE genes, is associated with repressive markers of transcription. However, as cells differentiate, the MIEP becomes associated with activatory marks of transcription concomitant with reactivation of IE gene expression (17–21). To date, a number of cellular transcription factors believed to be involved in recruitment of epigenetic modifiers to the MIEP during HCMV latency and reactivation have been identified (17–21). Some time ago, we identified the cellular transcription factor Yin Yang 1 (YY1) as a candidate repressor of the MIEP during latency (17). However, the importance of YY1 in regulating the MIEP in the context of latent infection was unclear, at least in the NT2D1 cell line (22), and attempts to generate YY1 knockout NT2D1 cells to directly determine if YY1 is required for latency in these cells were thwarted by cell death as a result of YY1 knockdown (23). YY1, though, has a number of characteristics which support a role for its involvement in regulation of the MIEP during latency and reactivation. Studies have shown that YY1 can bind to the MIEP and repress its activity, likely by recruitment of histone deacetylases, during latent infection of undifferentiated cell lines (17). Similarly, YY1 overexpression appears to prevent myeloid differentiation (24–26), which is crucial for HCMV reactivation. Interestingly, differentiation in some cell types has been shown to be mediated, at least in part, by decreases in YY1 due to an upregulation of the microRNA (miRNA) hsa-miR-29a (27), as YY1 can be directly targeted by this miRNA (28).

Confoundingly, tumor growth factor beta (TGFbeta) signaling can also lead to a SMAD-dependent upregulation of hsa-miR-29a (29, 30), and previous analyses of proteins secreted from HCMV latently infected CD34^+^ cells has shown that latent infection results in the induction of secreted TGFbeta (31), in part resulting from latency-associated expression of viral miRNA miR-US5-2 (32). Yet latently infected CD34^+^ cells, expressing high levels of TGFbeta, can be maintained in culture for extensive periods, suggesting that the ability of TGFbeta to activate hsa-miR-29a, degrade YY1, and drive differentiation must, during latency at least, be inhibited.

In this study, using genetic and pharmacological methods, including an iPSC model of HCMV latency, we identified bone morphogenetic protein receptor type 2 (BMPR2) as having a critical role in the maintenance of HCMV latent infection by modulating TGFbeta signaling. We showed that BMPR2 signaling induces SMAD6 via SMAD1/5/8. SMAD6 then suppresses TGFbeta signaling through TGFbeta receptor (TGFbetaR); suppression of TGFbeta signaling downregulates SMAD2 activation (33) and the expression of hsa-miR-29a. This, in turn, leads to a stabilization of YY1, leading to MIEP repression and the maintenance of latency. We further confirmed the critical role of YY1 in HCMV latency by its removal by CRISPR/Cas9 technology in THP1 cells and iPSCs. This prevents these cells from being able to support latent infection, instead leading directly to high levels of major IE expression. Interference with BMPR2 signaling leads to increased IE gene expression in otherwise latently infected cells, which then results in these cells becoming visible to T cell-mediated killing, thereby reducing the latent virus reservoir, and thus may be a potential therapeutic to reduce HCMV reactivation-mediated disease.

## RESULTS

### YY1 is required for HCMV latency in undifferentiated iPSCs and THP1 myelomonocytic cells.

**FIG 1:**
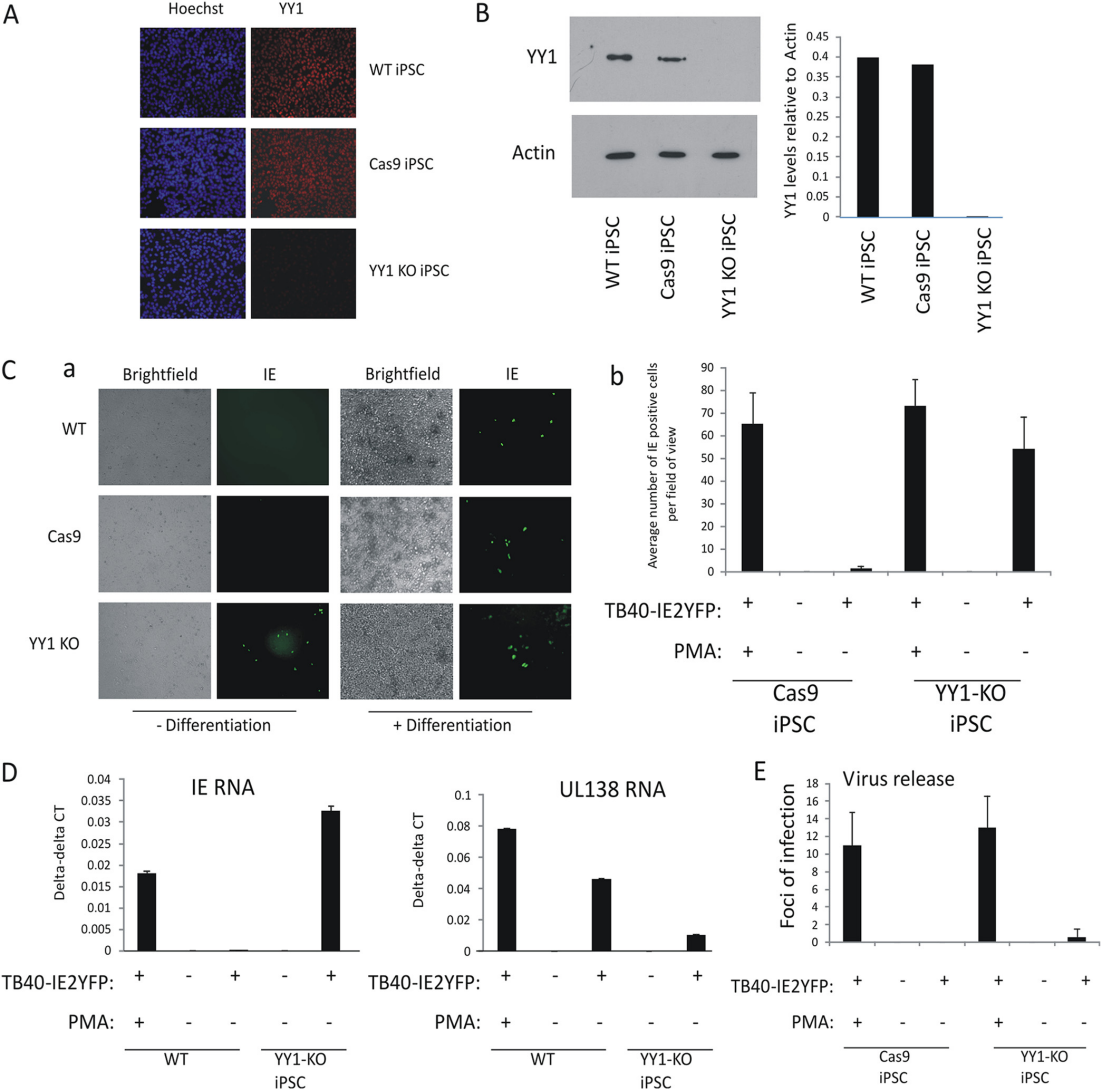

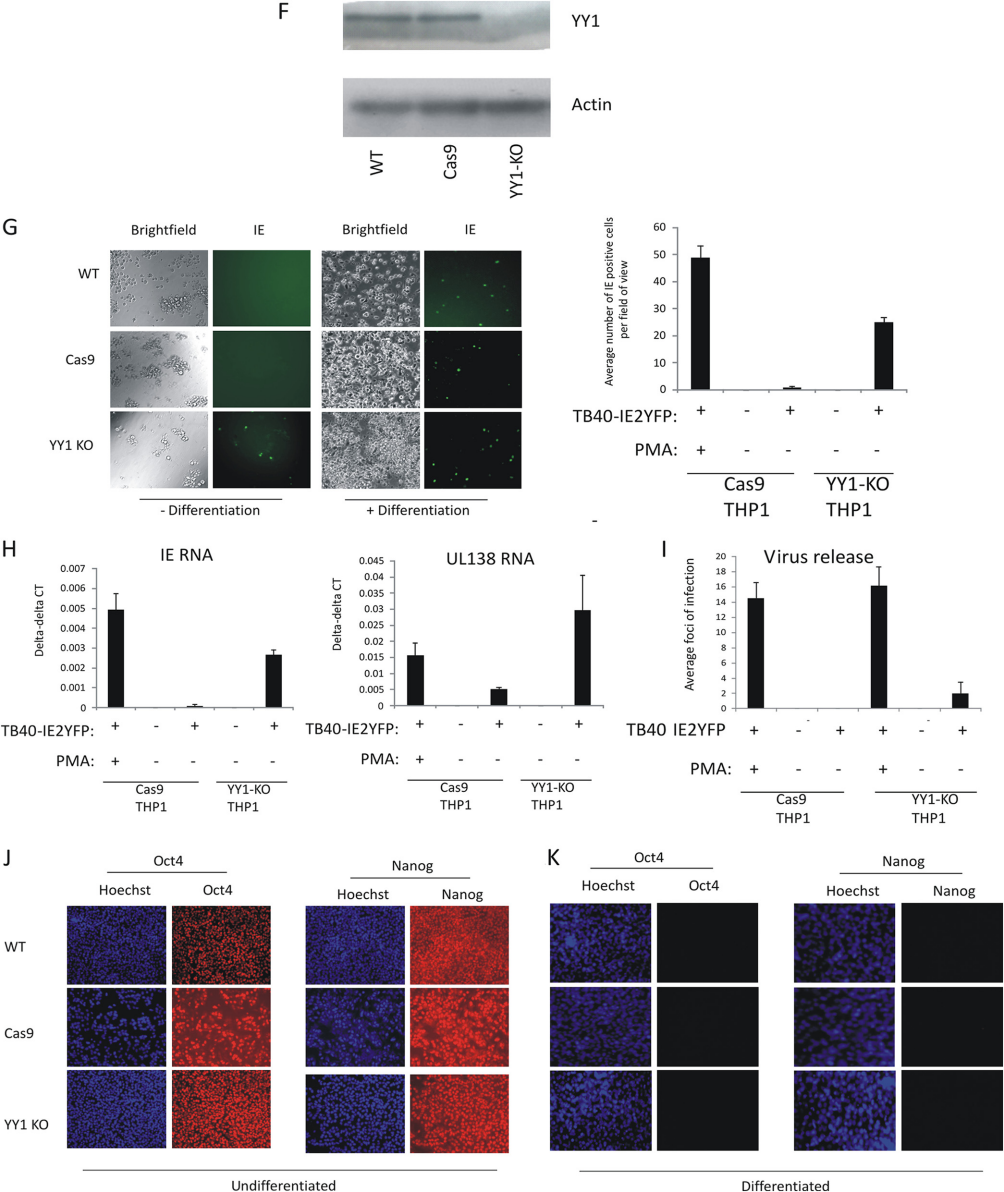

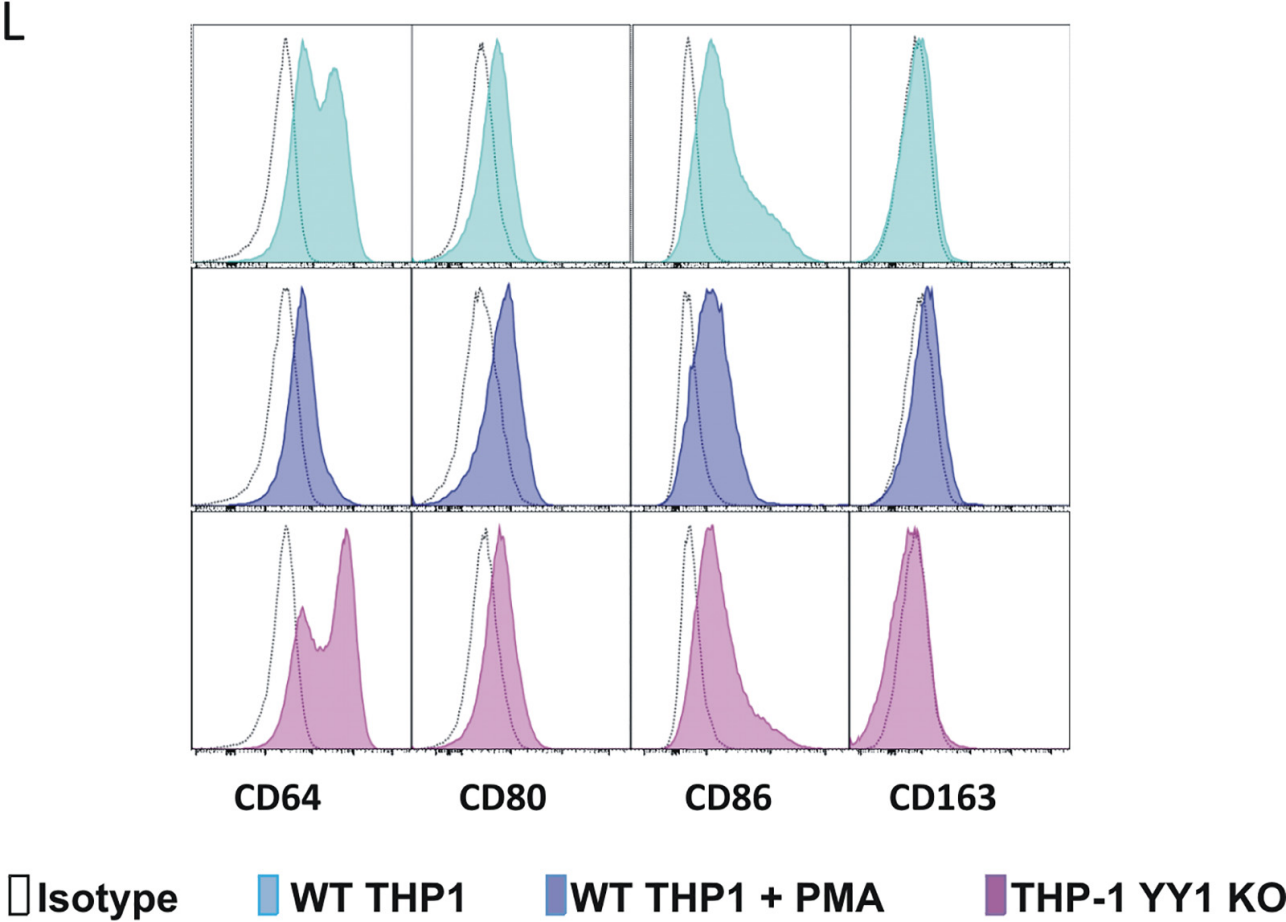
Cellular YY1 is required for HCMV latency. Wild-type iPSCs (WT-iPSCs), iPSCs which overexpressed Cas9 (Cas9-iPSCs), or which overexpressed Cas9 and were treated by CRISPR to remove YY1 (YY1-KO iPSCs) were stained for YY1 protein by indirect immunofluorescence staining (red), and the nuclei were costained with Hoechst (blue) (A). Alternatively, lysates from the same cells were analyzed by Western blotting for actin and YY1 proteins (B). (C) Undifferentiated iPSCs (- Differentiation) described for panels A and B) were infected with TB40E-IE2YFP and 4 days later analyzed by fluorescence for the presence of IE expression (green). This also included cells treated with PMA to induce differentiation (+ Differentiation) as a positive control for HCMV permissiveness. Bright-field images are also shown. Studies whose results are shown in panel C were performed in triplicate and enumerated (graph in panel C). Cells described for panel C were also harvested for RT-qPCR analysis and analyzed for IE expression and UL138 RNA relative to the housekeeping GAPDH gene (D). The supernatants from panel C were also transferred to fresh fibroblasts, and IE2-YFP-positive foci of infection in triplicate wells were counted after 9 days and enumerated (E). Wild-type THP1 cells (WT), THP1 cells which overexpressed Cas9 (Cas9), or which overexpressed Cas9 and a guide RNA to YY1 (YY1-KO) were harvested and analyzed by Western blotting for the housekeeping actin gene or YY1 as indicated (F). The experiment carried out for panel C was repeated using the THP1 cells described for panel F, and results are presented with graphical enumeration (G). The THP1 cells described for panel G were also harvested for RT-qPCR analysis of viral IE and UL138 genes alongside the housekeeping GAPDH gene (H). The supernatants from panel G were transferred to fresh fibroblasts, and IE2-YFP foci of infection in triplicate wells were counted after 9 days (I). The iPSCs described for panel A were also stained for the pluripotency markers Oct4 and Nanog (red) alongside a nuclear stain, Hoechst (blue) as indicated (J). The cells described for panel J were also differentiated prior to staining for Oct4 and Nanog (K). Finally, THP1 cells described for panel F were analyzed for the indicated differentiation markers by FACS (L). All graphs represent average values with standard deviation error bars.

In the NT2D1 cell line model of differentiation-dependent repression of the HCMV IE gene expression, the transcription factor YY1 is known to be able to bind to the enhancer region of the MIEP (17) and suppress MIEP activity in transfection assays, likely by recruitment of histone deacetylases (18–20). However, a direct role for YY1 in the suppression of IE gene expression during virus infection of undifferentiated NT2D1 cells has never been fully established; the key experiment of removing YY1 from these cells to assess their ability to support latent infection is confounded by cell death in YY1 knockout cells (23). Consequently, we used CRISPR/Cas9 technology to gene edit YY1 in iPSCs, a cell type, and derivatives thereof, which we have recently shown can be used as a tractable model of latency and reactivation in the myeloid lineage (14). Figure 1A shows that wild-type iPSCs stably expressing Cas9 and transfected with guide RNAs that target YY1 (YY1-KO iPSCs) have drastically reduced levels of YY1 expression by indirect immunofluorescence compared to isogenic iPSCs, which either stably express Cas9 alone (Cas9-iPSCs) or are nonmodified (wild type; WT-iPSCs). We confirmed this observation by Western blotting and densitometric analysis of YY1 (Fig. 1B). In contrast to NT2D1 cells (23), iPSCs with YY1 deleted were viable, arguing that YY1 is not essential for growth of these iPSCs. Next, we assessed whether YY1-KO iPSCs were able to establish latency. To do this, we infected WT-iPSCs, Cas9-iPSCs, and YY1-KO iPSCs with an IE2-yellow fluorescent protein (YFP)-tagged virus, which identifies cells undergoing IE gene expression. Without differentiation, IE2-YFP expression was not observed in WT- and Cas9-iPSCs but was clearly observed in YY1-KO iPSCs, and this was equivalent to levels observed in differentiated WT-iPSCs (Fig. 1C, panel a; a graphical representation of these data is shown in Fig. 1C, panel b). This was validated using reverse transcription-quantitative PCR (RT-qPCR) (Fig. 1D), which also showed that infected YY1-KO iPSCs had levels of IE RNA comparable to levels observed in infected WT-iPSCs which had been differentiated with phorbol myristate acetate (PMA); this was in contrast to the case with infected undifferentiated WT-iPSCs, which showed easily detectable levels of UL138 expression in the relative absence of IE, a characteristic of latent infection. Although IE expression was clearly induced in these YY1-KO iPSCs, there was little evidence of virus release compared to the case with PMA-differentiated cells (Fig. 1E), arguing that YY1 knockdown permits IE expression but not full virus reactivation. It was not possible to validate the CRISPR-edited YY1 cells by DNA digest because they were generated from random in-frame deletions. However, to ensure that this lack of latency establishment was due to disruption of YY1 expression, we alternatively used short hairpin RNA (shRNA)-YY1 lentiviral transduction to generate new YY1-KO cells which would have specifically targeted YY1. The same phenotype was observed in these cells, which suggests that the inability of YY1-KO cells to establish latency is specifically due a disruption of YY1 expression rather than, e.g., a nonspecific event brought about by gene editing (see Fig. S1A and B in the supplemental material). The removal of YY1 did not affect the ability of the cells to initially be infected since equivalent levels of infectivity were obtained when TB40-SV40GFP virus, which expresses green fluorescent protein (GFP) from the simian virus 40 (SV40) promoter, was used to infect WT-iPSCs, YY1-KO iPSCs, or shRNA-YY1-KO iPSCs (Fig. S1C). Similarly, there was no significant change in the ability of YY1-KO cells to be infected with HCMV after they had been differentiated, as determined by levels of IE gene expression after infection or virus release after coculture on indicator fibroblasts (Fig. S1D).

10.1128/mBio.00227-21.1 FIG S1 YY1 can be depleted from iPSCs using shRNA lentiviral vectors as well as by CRISPR/Cas9 editing which shows that YY1 is required for IE suppression in undifferentiated cells without affecting HCMV infectivity. WT-iPSCs, Cas9 iPSCs, CRISPR YY1 iPSCs, and shYY1 iPSCs were harvested for Western blot analysis of actin and YY1 proteins (A). iPSCs were treated with shRNA lentiviral vectors to beta2 microglobulin (shB2mic) or YY1 (shYY1), infected with TB40E-IE2YFP virus, and then either left undifferentiated or differentiated to induce IE gene expression and analyzed by bright-field and fluorescence microscopy (B). The indicated cells were infected with TB40-SV40GFP and then enumerated 3 days later (C). Finally, iPSCs and THP1 cells were differentiated with PMA for 48 h prior to infection with TB40E-SV40GFP and then immediately analyzed for numbers of cells positive for IE gene expression or assayed for virus production after subsequent coculture on indicator fibroblasts for 7 days. Graphs represent average values with standard deviations (D). Download FIG S1, PDF file, 0.1 MB. Copyright © 2021 Poole et al. 2021 Poole et al. https://creativecommons.org/licenses/by/4.0/ This content is distributed under the terms of the Creative Commons Attribution 4.0 International license.

### The removal of BMPR2 leads to an increase in sensitivity to TGFbeta.

YY1 can play a key role in transcriptional programming associated with cellular differentiation (24, 34, 35). One mechanism by which the cell is able to regulate expression of YY1 is via cytokine signaling, for example, TGFbeta. YY1 is also known to be targeted by cellular microRNAs (miRNAs) such as miR-29a (27), which, intriguingly, has been shown to be activated by TGFbeta (29, 30). Previous analyses have also shown that latent infection of myeloid progenitor cells with HCMV leads to induction of secreted TGFbeta, which acts to suppress T cell effector functions against the latently infected cell (31, 32). These observations, however, would argue that latently infected cells should be more prone to reactivation due to YY1 suppression via the TGFbeta/miR-29a axis. Yet, despite the functionally active levels of TGFbeta secretion induced by latency, myeloid progenitors can support long-term latent infection in culture (4, 36). We therefore wanted to interrogate how the YY1 axis was still able to suppress MIEP activity in a microenvironment containing high levels of TGFbeta. To do this, we again employed our iPSC model of HCMV latency (14).

**FIG 2:**
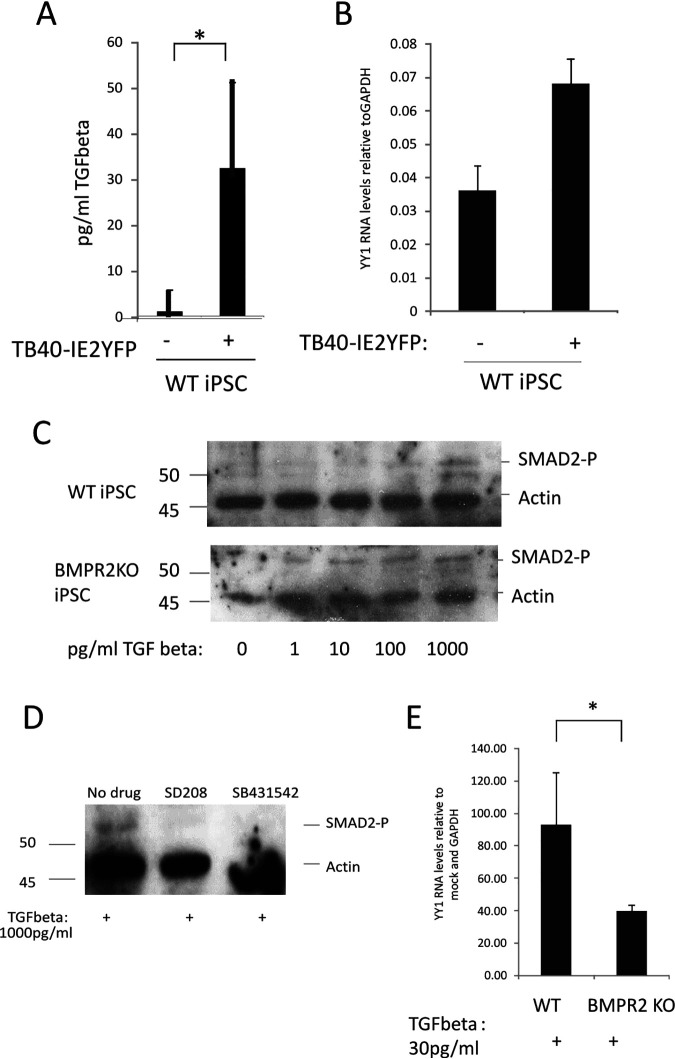
Latency-induced levels of TGFbeta stimulate SMAD2 phosphorylation, leading to a decrease in YY1 levels, only in the absence of BMPR2. iPSCs were either infected with TB40E-IE2YFP or left uninfected for 4 days before supernatants were harvested and analyzed for TGFbeta content by ELISA (A). Cells described for panel A were also analyzed by RT-qPCR for cellular YY1 transcript relative to the housekeeping GAPDH gene (B). Recombinant TGFbeta was added to either the WT-iPSCs (top) or BMPR2 KO-iPSCs (bottom) at the indicated concentrations for 1 h before harvesting for Western blotting for SMAD2 phosphorylation and the housekeeping actin gene (C). CD14^+^ monocytes were pretreated with SD208 (SD) or SB431542 (SB) for 30 min before addition of recombinant TGFbeta. Cells were then harvested for Western blotting for phosphorylated SMAD2 or the housekeeping actin gene (D). Finally, WT- or BMPR2 KO-iPSCs were treated with 30 pg/ml of TGFbeta for 1 h before harvesting by RT-qPCR for cellular GAPDH and YY1 genes as indicated (E). All graphs represent average values with standard deviations. Statistical significance was determined using Student’s *t* test.

We started by checking that latent infection of undifferentiated WT-iPSCs induced TGFbeta, as we have previously observed for CD34^+^ progenitors cells (31). Figure 2A shows that WT-iPSCs latently infected with HCMV did, indeed, show increased levels of TGFbeta (approximately 30 pg/ml) yet still expressed good levels of YY1 RNA (Fig. 2B). On this basis, we reasoned that despite the presence of TGFbeta in their secretome, these latently infected cells were unable to respond to TGFbeta at least with respect to suppression of YY1 expression.

It is well established that signaling through TGFbetaR can be modulated by signaling through other receptors, such as BMPR2, and a number of reports have addressed such cross talk between the TGFbeta and BMPR2 signaling pathways by mechanisms which include regulation of inhibitory signaling factors such as SMAD6 and -7; for example, BMPR2 signaling via SMAD1, -4, -5, or -8 can drive expression of SMAD6, which can inhibit TGFbeta signaling (37). Consistent with this, there are also reports which suggest that patients who have mutated BMPR2 receptors suffer from pulmonary hypertension due to their increased sensitivity to TGFbeta (38).

Analysis of the ability of WT-iPSCs to respond to TGFbeta, as assessed by the levels of SMAD2 phosphorylation, showed that treatment of WT-iPSCs resulted in increased SMAD2 phosphorylation only when high levels of TGFbeta were used (Fig. 2C). As it has also previously been shown that BMPR2 can suppress TGFbeta signaling and that removal of the BMPR2 receptor in endothelial cells can lead to an increase in responsiveness to TGFbeta (33), we analyzed the TGFbeta responsiveness of iPSCs in which *BMPR2* had been knocked out by CRISPR/Cas 9 technology (BMPR2 KO-iPSCs) (39). The removal of BMPR2 by this method does not change infectivity of the cells (14). Figure 2C shows that in contrast to WT-iPSCs, which require high levels of TGFbeta to elicit SMAD phosphorylation, BMPR2 KO-iPSCs are sensitive to TGFbeta at concentrations as low as 1 pg/ml. These data suggest that following the removal of BMPR2, these iPSCs become more sensitive to TGFbeta, leading to signaling via TGFbetaR to induce the phosphorylation of SMAD2. As a final verification that the removal of BMPR2 leads to increased sensitivity to TGFbeta signaling via SMAD2, we then used the drugs SB431542 and SD208, which are known to inhibit TGFbeta signaling at the receptor. The two different TGFbeta-targeting drugs were used to control for any off-target side effects. If the BMPR2 receptor was removed, we would predict that in the presence of drugs specific for the TGFbeta signaling pathway would no longer lead to an increase of SMAD2 signaling in these cells in response to TGFbeta. Figure 2D shows that activation of the TGFbeta receptor with TGFbeta can be blocked using specific TGFbeta inhibitors, SB431542 and SD208, since in the presence of these drugs TGFbeta did not induce SMAD2 phosphorylation. Finally, the addition of TGFbeta at 30 pg/ml (levels observed in latently infected WT-iPSCs) led to a reduction in levels of YY1 in BMPR2 KO-iPSCs but not WT-iPSCs (Fig. 2E). Taken together, these data suggest that in the absence of BMPR2, cells become more sensitive to TGFbeta-mediated signaling via SMAD2 and this is correlated with a decrease in levels of YY1.

### Removal of BMPR2 leads to an inability of cells to establish and maintain HCMV latency.

**FIG 3:**
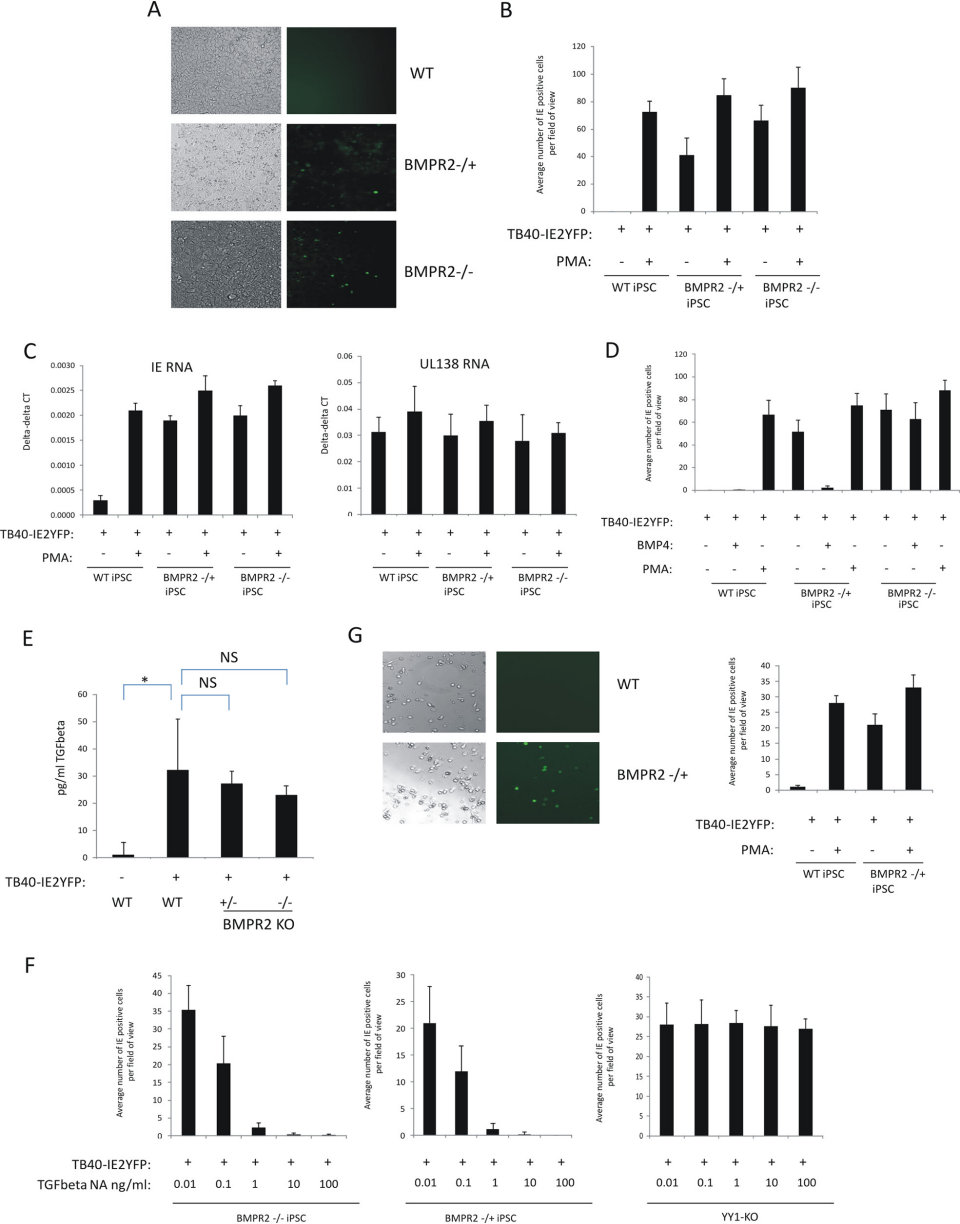
Latency cannot be established in the absence of BMPR2 but can be recovered if TGFbeta is also removed using neutralizing antibodies. WT-iPSCs or BMPR2^−/+^ or BMPR2^+/+^ iPSCs, as indicated, were infected with TB40E-IE2YFP for 4 days before analysis by immunofluorescence (IF; (right) and bright-field microscopy (left) (A). Cells from panel A were enumerated (10 fields of view of 100 cells each) alongside PMA-treated positive controls, and graphs represent triplicate experiments (B). Cells from panel B were also harvested for RNA analysis for the presence of viral lytic IE gene (left graph) and latency-associated gene UL138 (right graph) relative to the housekeeping GAPDH gene (C). WT-iPSCs and BMPR2^−/+^ and BMPR2^−/−^ iPSCs, as indicated, were pretreated with BMP4 (50 ng/ml) prior to infection with TB40E-IE2YFP. IE2-YFP-positive cells were then enumerated in triplicate with 10 fields of view of 100 cells each (D). WT-iPSCs and BMPR2^−/+^ and BMPR2^−/−^ iPSCs were infected with TB40E-IE2YFP for 4 days before supernatants were assayed for secreted TGFbeta by ELISA. Uninfected WT cells are also shown, and statistical significance determined using Student’s *t* test (E). Finally, BMPR2^−/−^ and BMPR2^−/+^ or YY1-KO iPSCs, as indicated, were pretreated with the indicated concentrations of TGFbeta neutralizing antibody before infection with TB40E-IE2YFP. Medium was changed with fresh neutralizing antibody daily and IE2-YFP-positive cells were scored by IF after 4 days. Triplicate experiments were carried out with 10 fields of view of 100 cells each (F). WT and BMPR2^−/+^ cells were differentiated along the myeloid lineage into monocyte-like cells and then either infected with TB40E-IE2YFP for 4 days before analysis by IF (right) and bright-field microscopy (left) as well as being enumerated alongside a PMA-positive control in triplicate with 10 fields of view of 100 cells each (right), with standard deviation error bars shown (G). All graphs show mean values with standard deviations.

Given that removing BMPR2 from iPSCs causes them to respond to levels of TGFbeta that are routinely observed in supernatants of latently infected iPSCs, and that it results in reduced levels of YY1, we next tested whether BMPR2 KO-iPSCs are able to establish and maintain latent infection. To test this, three different cell types were compared: WT-iPSCs, heterozygous *BMPR2* iPSCs, and homozygous deletion *BMPR2* iPSCs. Figure 3A shows that while WT-iPSCs were able to establish and maintain latent HCMV infection, cells which were either heterozygous or homozygous for the *BMPR2* deletion were unable to support latency. These results are shown numerically in Fig. 3B, which also shows that the levels of IE2-YFP expression after infection of cells heterozygous or homozygous for the *BMPR2* deletion were close to levels observed if the cells had been differentiated with PMA. Additionally, Fig. 3C shows that while the latency-associated gene UL138 was expressed in all undifferentiated and differentiated infected cell types as expected (since UL138 is also expressed during lytic infection), lytic IE gene expression was clearly repressed in undifferentiated WT-iPSCs but expressed to high levels in infected iPSCs which lacked BMPR2. As it is known that BMPR2 levels can be restored in heterozygous *BMPR2*-negative cells by treatment with exogenous BMP4, likely by inducing *BMPR2* expression from the heterozygous gene copy (39), we reassessed the ability of heterozygous *BMPR2*-deleted iPSCs to support latency upon treatment with BMP4. Figure 3D shows that incubation of heterozygous *BMPR2*-deleted iPSCs with exogenous BMP4 restored the ability of these cells to support a latent HCMV infection, which was not the case for homozygous *BMPR2*-deleted iPSCs treated with BMP4.

A more trivial explanation for the inability of BMPR2 KO-iPSCs to establish and maintain latency could be that these cells simply expressed higher levels of TGFbeta, which, in turn, caused downregulation of YY1. To address this, enzyme-linked immunosorbent assays (ELISAs) were carried out on the secretomes from the infected WT-iPSCs to assess levels of TGFbeta induction during infection. Figure 3E shows that levels of induction of TGFbeta were not statistically different after infection of *BMPR2* WT, heterozygous or homozygous knockout iPSCs, ruling out that the differences in permissiveness of these cells were due to differences in levels of TGFbeta induction. However, if TGFbeta was neutralized during infection of BMPR2 KO-iPSCs, the latent phenotype could be rescued (Fig. 3F, left and middle graphs). This was specific for BMPR2 deficiency since removal of YY1 could not be rescued by the removal of TGFbeta (Fig. 3F, right graph), suggesting that BMPR2 acts upstream of YY1. Taken together, these data suggest that the presence of BMPR2 is required for a latent infection to be established, as it prevents the infected cells from decreasing YY1 in response to the latency-associated increase in TGFbeta.

We also wanted to confirm these observations in monocytes. Consequently, we generated monocytes from WT- or BMPR2 KO-iPSCs, as we have done previously (14), and repeated these analyses. Figure 3G confirmed our observations using infected iPSCs in that monocytes derived from heterozygous *BMPR2* deletion iPSCs were also unable to support HCMV latency, whereas monocytes derived from WT-iPSCs established a latent infection.

### BMPR2 signaling to inhibitory SMAD6 is required for latency establishment.

Our data, so far, have shown that BMPR2 signaling plays a key role in establishing HCMV latency in progenitor cells as well as monocytes derived from them. Therefore, we next addressed what aspect of the BMPR2 signaling cascade was required for this. It is already known that patients with genetic mutations in *BMPR2* are more prone to pulmonary hypertension, and it has been suggested that this is because endothelial cells in these patients become more sensitive to TGFbeta (33, 38, 40). One proposed mechanism for this increased TGFbeta sensitivity is coreceptor competition such that loss of BMPR2 could result in increased levels of functional TGFbetaR due to coreceptor abundance and thus enhanced sensitivity to TGFbeta ligand. For example, Alk1 has been proposed to act as a coreceptor in both BMPR2 and TGFbeta signaling, which may be differentially regulated in the absence of BMPR2 (41, 42). Another possible mechanism for such a link between BMPR2 levels and responses to TGFbeta could be at the level of their signal transducers. For instance, BMPR2 is known to signal via SMAD1, -5, and -8 to drive expression of inhibitory SMAD6, which can downregulate signaling via TGFbeta (37). Therefore, a decrease in levels of BMPR2 could lead to a lack of inhibitory SMAD6 expression and a concomitant increase in sensitivity to TGFbeta.

**FIG 4:**
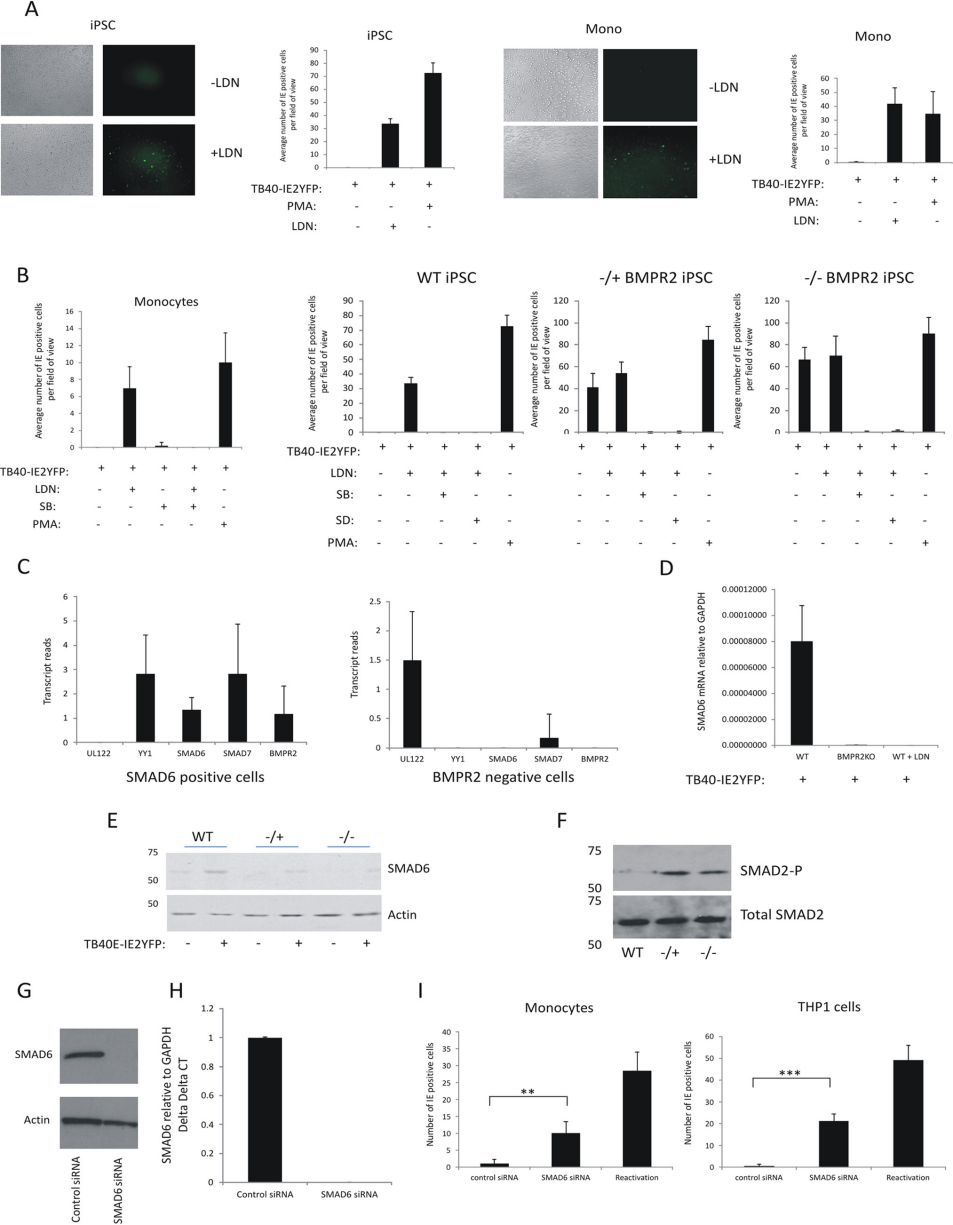
BMPR2 inhibitor LDN prevents the establishment of HCMV latency in iPSCs and monocytes, but this can be rescued with TGFbeta inhibitors and BMPR2-stimulated SMAD6 aids maintenance of latency. Monocytes or iPSCs, as indicated, were infected with TB40E-IE2YFP for 4 days in the presence or absence of LDN (medium was changed daily and replaced with fresh LDN). Cells were then analyzed by IF for IE2-YFP and bright-field microscopy (left) (A). These cells were enumerated alongside a PMA-positive control, and the graph represents triplicate experiments in 5 fields of view of 100 cells each (right) (A). Monocytes, WT-iPSCs, BMPR2^−/+^ iPSCs, or BMPR2^−/−^ iPSCs were infected with TB40E-IE2YFP in the presence or absence of LDN, SB431542, SD208, or PMA as indicated, with medium and drug changes every day for 4 days before enumeration of IE2-YFP-positive cells in 6 fields of view from 100 cells each from triplicate experiments. The graph shows mean values, with standard deviation error bars (B). iPSCs were infected with TB40E-GATA2mCherry for 4 days prior to sorting and analysis by single-cell RNA-seq analysis. Six cells were analyzed for numbers of transcripts of the UL122, YY1, SMAD6, SMAD7, and BMPR2 genes. Cells in the left graph were selected on the basis of the ability to detect SMAD6 transcript. Cells in the right graph represent cells which were BMPR2 negative. Graphs show mean values with standard deviation error bars (C). BMPR2^−/−^ iPSCs and WT-iPSCs were infected with TB40E-IE2YFP and then either treated with LDN or left untreated as indicated prior to harvesting for RNA analysis. Levels of GAPDH and SMAD6 were determined by RT-qPCR. The data are from triplicate samples, and average delta delta *C_T_* values for SMAD6 relative to GAPDH are shown with standard deviation error bars. WT-iPSCs, BMPR2^−/−^ iPSCs, or monocytes that had been treated with LDN were harvested for RT-qPCR analysis of SMAD6 RNA relative to the housekeeping GAPDH gene (D). WT-iPSCs and BMPR2^−/+^ and BMPR2^−/−^ iPSCs were either left uninfected or infected with TB40E-IE2GFP for 4 days before harvesting for Western blotting and analysis for the presence of SMAD6 and actin proteins (E). The latently infected cells described for panel E were analyzed for total SMAD2 and phosphorylated SMAD2 by Western blotting (F). THP1 cells were nucleofected with an siRNA to SMAD6 and then left for 48 h before analysis of SMAD6 and actin proteins by Western blotting (G) and RNA by RT-qPCR; the graph represents delta delta *C_T_* with standard errors shown (H). Finally, CD14^+^ monocytes or THP1 cells were infected with IE2YFP-TB40E and left for 3 days (I). After 3 days, the cells were nucleofected with control siRNA or SMAD6 siRNA or, as a control, reactivated with GM-CSF/interleukin 4 (IL-4) and lipopolysaccharide (LPS) (I, left) or PMA (I, right). Cells were then enumerated for IE positivity. The graph represents 6 fields of view of 100 cells, with standard deviations and significance determined with Student’s *t* test (I).

To analyze the role of BMPR2 signaling in maintaining latency, the BMPR2 inhibitor LDN 193189 (LDN), which targets BMPR2 coreceptors Alk1/2/3 (43), was used to test whether cells in which BMPR2 is inhibited by this drug could still establish HCMV latency. Figure 4A shows that treatment of undifferentiated WT-iPSCs or CD14^+^ monocytes with this BMPR2 inhibitor prevented them from being able to establish latency. Because LDN targets Alk1 (in addition to Alk2 and -3), which can be used by either TGFbetaR or BMPR2 signaling, the mechanism by which BMPR2 regulates HCMV latency is unlikely to be due to reallocation of the Alk1 receptor to the TGFbetaR. Indeed, the use of the TGFbetaR-blocking drug SD208, which specifically blocks Alk5 signaling, showed that primary CD14^+^ monocytes can still maintain HCMV latency (Fig. 4B). Additionally, in cells in which BMPR2 was inhibited either by gene editing or biochemically (using LDN), additional blocking of TGFbetaR by SD208 rescued the ability of these BMPR2 signaling-inhibited cells to establish a latent infection. These data suggest that it is likely that BMPR2 works antagonistically with the TGFbetaR with respect to maintenance of latency.

These data suggest that the BMPR2 signaling pathway is important in the establishment of HCMV latency by reducing signaling by TGFbeta, which is secreted to high levels in latently infected cells. Consequently, we reasoned that this could be because the TGF-inhibitory SMADs, such as SMAD6, which is expressed in response to BMPR2 signaling, are poorly expressed in the absence of BMPR2. Initially, to analyze whether SMAD6 might be involved in the establishment of latency, we reassessed previously published single-cell RNA sequencing (RNA-seq) data of latently infected iPSCs (14). While reads of cellular genes were too low in the original analyses to be statistically significant, in general, cells which were positive for SMAD6 transcripts were also positive for RNAs for SMAD7, YY1, and BMPR2 but negative for UL122 RNA (a major IE viral lytic gene). In contrast, infected BMPR2 KO-iPSCs, which were IE transcript positive, contained little detectable SMAD6 (Fig. 4C). These indicative observations would be consistent with the view that SMAD6 and YY1 may play a role in repression of IE gene expression in undifferentiated myeloid cells. To test more thoroughly whether SMAD6 was important for establishment of latency, WT or *BMPR2* knockout cells that had been infected with HCMV were analyzed by RT-PCR for the presence of SMAD6 (Fig. 4D). BMPR2 KO-iPSCs expressed significantly less SMAD6 than did WT-iPSCs, consistent with the view that the presence of BMPR2 can lead to the expression of SMAD6 in latently infected cells. As we would have predicted, treatment of iPSCs with LDN, conditions in which iPSCs become able to allow IE gene expression after infection (fig. 4B), also led to a decrease in SMAD6 mRNA expression (Fig. 4D). Additionally, Western blot analysis showed that SMAD6 protein was also decreased in iPSCs edited for *BMPR2* (Fig. 4E). One reported effect of the removal of inhibitory SMAD6 is an activation of SMAD2. Consistent with this, when iPSCs were infected with HCMV, SMAD2 phosphorylation was detected at significantly higher levels in iPSCs edited for BMPR2 (Fig. 4F). To test whether SMAD6 plays a role in HCMV latency, cells were treated with small interfering RNA (siRNA) to SMAD6. The siRNA was effective at reducing both the protein and RNA levels (Fig. 4G and H). Figure 4I shows that for both CD14^+^ monocytes and THP1 cells, removal of SMAD6 renders the cells significantly less capable of maintaining latency. Taken together, these data are consistent with the view that in order to support a latent infection, BMPR2 is required in order to express inhibitory SMAD6, which then prevents TGFbeta receptor signaling.

### iPSCs lacking BMPR2 have increased levels of hsa-miR-29a, which correlates with decreased levels of YY1 and decreased YY1 occupancy on the viral MIEP.

**FIG 5:**
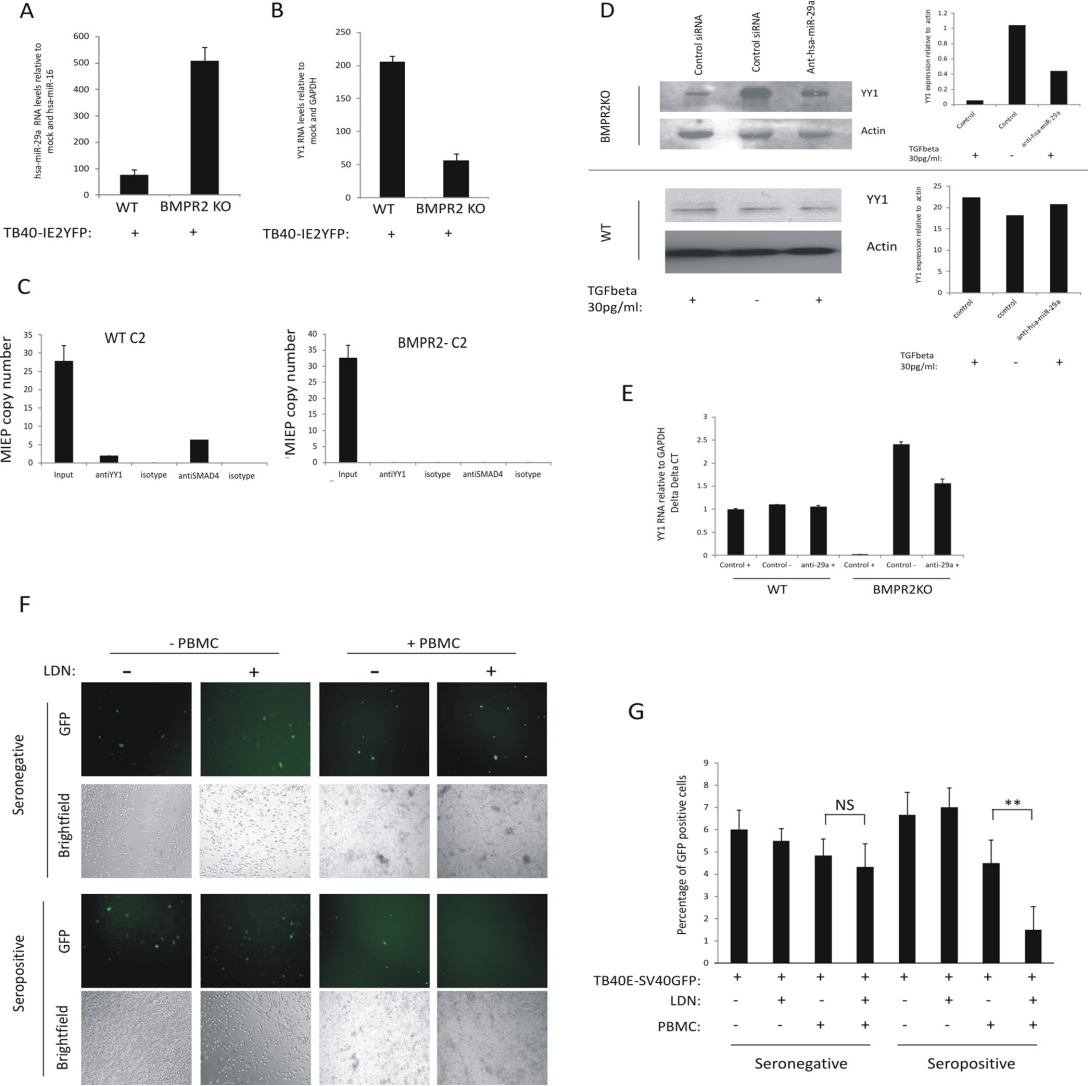
The absence of BMPR2 leads to a decrease in levels of YY1 mRNA during HCMV latency and a lack of YY1 occupancy on the MIEP. WT-iPSCs and BMPR2^−/−^ iPSCs were latently infected with HCMV for 4 days before analysis for miRNA hsa-miR-29a and the housekeeping miRNA hsa-miR-16. Graphs represent triplicate mean delta delta *C_T_* values with standard deviation error bars (A). WT-iPSCs and BMPR2^−/−^ iPSCs were either left uninfected or infected with TB40E-IE2YFP for 4 days before the RNA was harvested and analyzed for YY1 and housekeeping gene GAPDH mRNAs (B). WT-iPSCs and BMPR2^−/−^ iPSCs were infected with TB40E-IE2YFP for 4 days before harvesting for ChIP analysis to detect YY1 association with the MIEP. Input samples were diluted 1:10, and the MIEP was detected by qPCR (C). Graphs represent triplicate mean values with standard deviation error bars. Finally, BMPR2 KO cells or WT cells (D and E) were transfected with control siRNA (control) or an antagomir to hsa-miR-29a (anti-hsa-miR-29a) in the presence or absence of 30 pg/ml of TGFbeta. After 48 h, cells were harvested for Western blotting (left, D) for actin and YY1 and bands were then analyzed by densitometry (right, D). Alternatively, cells were harvested for RNA. (E) Analysis of YY1 and GAPDH transcripts. The graph represents delta delta *C_T_*, with standard deviations shown. Finally, CD14^+^ monocytes were isolated from an HCMV-seronegative or HCMV-seropositive individual and remaining PBMCs were stored. The CD14^+^ cells were then infected with TB40E-SV40GFP and latency was established for 3 days. After this time, LDN was added to the cells as indicated for 24 h before addition of PBMCs at physiological effector-to-target (E:T) ratios (F and G). After 24 h, cells were analyzed by light and fluorescence microscopy (F) and GFP-positive cells were enumerated and presented graphically; standard deviation error bars are shown from 6 fields of view (**, significance value of <0.005 by Student’s *t* test; NS, not significant) (G).

We wanted to address how other known effects of increased TGFbeta signaling, which might be predicted to impact detrimentally on latent carriage, were counteracted during latency. One other established effect of TGFbeta signaling is the targeting of YY1 due to upregulation of the cellular miRNA hsa-miR-29a (28). Therefore, we analyzed levels of hsa-miR-29a in BMPR2 KO-iPSCs and WT-iPSCs during latent infection. Figure 5A shows that hsa-miR-92a was significantly upregulated in BMPR2 KO-iPSCs, consistent with the low levels of YY1 in these cells. Consequently, we next tested whether this decrease in YY1 in BMPR2 KO-iPSCs leads to a reduction in YY1 binding to the viral MIEP. Figure 5B shows that in the absence of BMPR2, the decreased levels of YY1 did indeed result in lower occupancy of YY1 on the viral MIEP (Fig. 5C). Consistent with this, hsa-miR-29a affected YY1 protein in response to 30 pg/ml of TGFbeta, but only in the absence of BMPR2 (Fig. 5D), and this correlated with YY1 RNA levels, as expected (Fig. 5E). Together, these data show that BMPR2 is required for SMAD6-mediated repression of TGFbeta signaling during HCMV latency. In the absence of BMPR2, latency-associated increases in TGFbeta stimulate SMAD2 phosphorylation, which, in turn, drives expression of hsa-miR-92a, which degrades YY1 and relieves repression of MIEP-driven IE expression.

Finally, as a proof of principle for a therapeutic application of our observations, we asked whether transient induction of IE gene expression in experimentally latent primary monocytes could make these cells become targets for HCMV-specific T cells. To determine this, we infected primary monocytes from an HCMV-seropositive donor with TB40E-SV40GFP virus (which expresses GFP from the SV40 promoter and therefore is not dependent upon IE gene expression for detection) in which we disrupted BMPR2 signaling chemotherapeutically with LDN and then exposed these cells to autologous peripheral blood mononuclear cells (PBMCs) (Fig. 5F and G). As expected, PBMCs from an HCMV-seronegative donor were unable to target and kill experimentally latent monocytes from that donor (Fig. 5F, top, and Fig. 5G). However, when monocytes and PBMCs from an HCMV-seropositive donor were used, experimentally latent monocytes which had received LDN to induce IE expression were killed at significantly higher levels than those which had not been treated with LDN (Fig. 5F, bottom, and Fig. 5G). This argues that transient induction of viral IE gene expression by BMPR2 inhibitors in otherwise latently infected cells makes them targetable by HCMV-specific immune cells which would not, otherwise, surveil and target this latent reservoir.

## DISCUSSION

It is critical to understand the mechanisms which establish and maintain HCMV latent infection, and then allow the progression to a lytic phase. Moreover, this understanding would help address an important unmet clinical need, particularly in the setting of solid-organ or stem cell transplants, in which HCMV reactivation can result in significant morbidity and mortality in the graft recipient. Previous analyses have shown that transient induction of IE gene expression in otherwise latently infected myeloid cells can result in their targeting and killing by resident HCMV-specific T cells already present in the seropositive donor (18). Consequently, BMPR2 inhibitors, such as LDN, could also be used to induce transient lytic viral gene expression in the hope of targeting and reducing the latent HCMV reservoir prior to stem cell transplantation.

**FIG 6:**
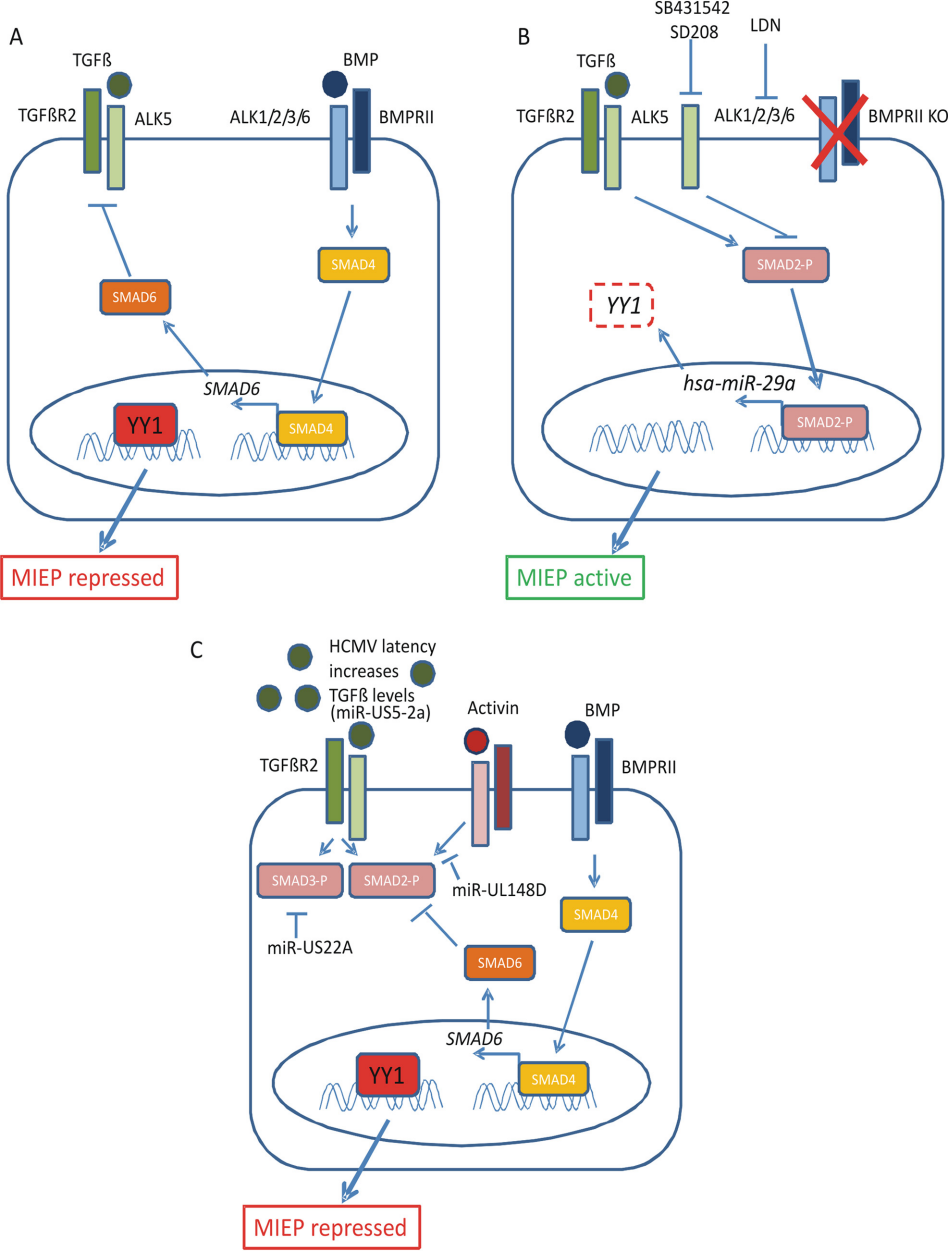
Mechanism for the role of BMPR2 in supporting HCMV latency and inhibition of SMAD2/3 signaling. In the presence of BMPR2 (A), there is expression of the inhibitory SMAD protein SMAD6. This prevents activation of the TGFbeta receptor by TGFbeta, which is secreted during latency, and therefore, the YY1 present in the cell is associated with the MIEP, enhancing the maintenance of HCMV latency. In the absence of BMPR2, by either direct removal of BMPR2 or inhibition of BMPR2 signaling using LDN (B), there is a lack of inhibitory protein SMAD6, and this allows the TGFbetaR to respond to the TGFbeta in the secretome, stimulating SMAD2/3 phosphorylation and leading to the expression of the miRNA hsa-miR-29a, which, in turn, causes degradation of the protein YY1. This protein is then unable to associate with the MIEP, and lytic IE gene expression can occur. The activation of lytic gene expression can be inhibited using specific inhibitors SB431542 and SD208. Finally, TGFbeta signaling is upregulated during latency (31) by viral miRNA miR-US5-2a (49), and the virus has a number of mechanisms in place to prevent TGFbeta- and activin-mediated SMAD2/3 signaling. These mechanisms include the expression of viral miRNAs US22A (49) and miR-UL148D (53) as well as the presence of BMPR2 (C).

A number of cellular factors have been implicated in the establishment and maintenance of HCMV latency. One of these, YY1, is known to associate with the viral MIEP and repress activity from the MIEP in certain cell types. However, the importance of YY1 in the context of latent virus infection has not been fully elucidated. The data presented in this paper show a mechanism by which HCMV latency in undifferentiated myeloid cells can be regulated by the cellular transcription factor YY1. During latency, the BMPR2 receptor signals to induce expression of the inhibitory protein SMAD6. In turn, SMAD6 prevents activation of the TGFbetaR by secreted TGFbeta, which is at high levels in the latency-associated secretome, which would otherwise result in decreased YY1 expression. In cells where BMPR2 is missing, SMAD6 is not expressed, allowing TGFbeta signaling to decrease YY1 RNA via the cellular miRNA hsa-miR-29, resulting in a lack of MIEP repression due to a decrease in YY1 protein levels. Essentially, in the absence of YY1, it is not possible to establish a latent infection in the monocytic cell line THP1 or in undifferentiated iPSCs (summarized in Fig. 6).

Our analysis also highlights the importance of the cellular receptor protein BMPR2 for the regulation of HCMV latency through the YY1/TGFbeta axis. The upregulation of TGFbeta during a lytic HCMV infection is well established as well as the virus-induced countermeasures to control TGFbeta signaling (44–46). For example, virus-induced TGFbeta has been shown to suppress granulocyte-macrophage colony-stimulating factor (GM-CSF) in osteosarcoma cells during lytic infection (47).

Our analyses have so far indicated the important role of YY1 in the establishment of HCMV MIEP suppression and latency. Since TGFbeta regulation appears to be critical in both latent and lytic phases of the HCMV life cycle, it would also be of interest to analyze the role of YY1 in reactivation by, for instance, the generation of a conditional YY1-KO cell line to allow latency to be established in the presence of YY1 and then analyze the effects of YY1 removal in a latently infected cell. Such studies are ongoing.

Clearly, from the data presented here, as well as from previous work, the upregulation of TGFbeta by a number of mechanisms appears to be important for latent HCMV carriage (31, 32, 48, 49). However, downstream signaling by this latency-associated increase in TGFbeta is also, subsequently, suppressed by BMPR2, which prevents TGFbeta-mediated SMAD2 signaling (this study), as well as by the viral miRNA miR-US22A, which targets SMAD3 (32) (summarized in Fig. 6).

A previous study on equine herpesvirus suggested that BMPR2 may play a role in the latency of this herpesvirus, but no mechanism was defined (50). Here, we show that with HCMV, cells cannot establish a latent infection in the absence of BMPR2 and that this is due to the inability of these cells to be refractory to the presence of cellular TGFbeta, which is known to be induced during latent infection. Sensitivity of cells to TGFbeta can be very cell type specific. One study suggests that the limit for sensitivity to TGFbeta is on the order of 25 ng/ml in epithelial cells (51). Therefore, it may not be surprising that the levels of secreted TGFbeta observed in cultured uninfected WT-iPSCs are too low to generate high enough levels of TGFbeta signaling to lead to SMAD2 phosphorylation in these cells but that this can occur when these levels of TGFbeta are increased by latent infection.

The removal of BMPR2 from iPSCs leading to increased TGFbeta sensitivity, reflected in SMAD2 phosphorylation, is consistent with observations in patients who have mutations in BMPR2 leading to the cells becoming more sensitive to secreted TGFbeta based on their level of SMAD2 phosphorylation. Studies of endothelial cells from patients lacking BMPR2 also suggest a balanced interplay between BMPR2 and TGFbeta signaling (33, 52). Similarly, drug studies using LDN and SD208, which target BMPR2 and TGFbetaR, respectively, have also shown that the two pathways (and drugs) can work antagonistically, consistent with the results obtained when using these inhibitors on latently infected cells. For instance, we can activate the MIEP and inhibit latency with the use of LDN, which blocks the BMPR2 signaling pathway, and then rescue the latent phenotype by using the TGFbetaR antagonist SD208. Similarly, it has been shown that signaling from one receptor can often downregulate the other (37). There have been various mechanisms proposed for this. It is possible that the abundance of Alk protein, a coreceptor for both BMPR2 and TGFbetaR, varies in its level of association with the BMPR2 and TGFbeta receptors. Alternatively, signaling from one pathway (e.g., BMPR2) can lead to inhibitory proteins, such as SMAD6 and SMAD7, being expressed, which then prevent signaling from the other pathway (e.g., TGFbeta). While we have not ruled out that coreceptor balance may play a role in HCMV latency, our data presented here suggest that a key component of this effect is the expression of SMAD6, which inhibits responsiveness of iPSCs to TGFbeta. These observations correlate well with a recent unpublished RNA-seq analysis of iPSC-derived endothelial cells (iPSC-ECs) in which SMAD6 was found to be significantly reduced in BMPR2 heterozygous iPSC-ECs compared to wild-type iPSC-ECs. A previous report (32) has also shown that a viral miRNA, HCMV miR-UL22A, which is expressed during latency, targets SMAD3 (which can act together with SMAD2), and it is possible that BMPR2 and miR-UL22A act together for optimal latency maintenance (summarized in Fig. 6).

In addition to TGFbeta, SMAD2 phosphorylation can also be activated by activins, and this can be mediated through the use of Alk proteins (such as Alk4) as coreceptors. Interestingly, we have previously shown that HCMV expresses a viral miRNA (HCMV UL148D) during latency which targets the activin receptor (53), which could also prevent activation of SMAD2 (summarized in Fig. 6). Consequently, inhibition of SMAD2 phosphorylation during latency likely results from a number of latency-associated mechanisms.

In contrast to WT cells, cells lacking BMPR2 respond to TGFbeta and one result of this was the decreased expression of YY1 in these cells. This correlates well with the known decreases in YY1 during differentiation of myeloid cells that lead to reactivation. Consistent with our observations, TGFbeta is known to result in degradation of YY1 directly via the miRNA hsa-miR-29a (29). Interestingly, our previous analyses (54) have shown that hsa-miR-29a is decreased during latent HCMV infection of CD34^+^ cells 1.5-fold, suggesting that latent infection may help maintain levels of YY1, although levels of YY1 were never assessed in that analysis.

One previous study analyzed the deletion of putative YY1 binding sites in the viral MIEP and concluded that at least in NT2D1 cells, YY1 binding to the MIEP was not required for repression of IE expression in this cell type (22). At present, the reason for this apparent discrepancy regarding a role for YY1 in MIEP repression is unclear. Possible explanations could lie in the previous use of the Towne isolate of HCMV, which is a laboratory-adapted strain often with deletions of a number of important HCMV functions associated with HCMV IE regulation. It is also possible that, at least in NT2D1 cells, YY1 is redundant for MIEP repression in the context of the virus. Regardless, our data argue strongly that in undifferentiated primary myeloid cells, HCMV latency is unable to be established in the absence of YY1.

The potential role of BMPR2 in the homeostasis of HCMV latency and reactivation could have implications in certain clinical settings. The drugs used in this study, which target the BMPR2 pathway and lead to T cell-mediated killing of HCMV latently infected cells, could have potential roles in the treatment of hypertension but also HCMV latency/reactivation *in vivo*. For instance, since such drug treatment transiently activates IE gene expression, in otherwise latently infected cells, this could be used for the basis of so-called “shock and kill” strategies to allow host targeting of the latent HCMV reservoir, as we have described for the use of histone deacetylase inhibitors (18). Alternatively, the TGFbeta pathway-inhibitory drugs, such as SB431542 and SD208, could be repurposed to maintain latency and hinder reactivation.

## MATERIALS AND METHODS

### Cells and viruses.

The iPSCs (WT, BMPR2^−/+^, and BMPR2^−/−^) have been described previously (39). THP1 cells were also cultured as described previously (8). The viruses used were derived from the clinical TB40E strain of HCMV. Three viruses were used which have been described previously: TB40E-IE2YFP, which expresses YFP fused to the immediate early gene IE2 (IE86), TB40E-GATA2mCherry, and TB40E-SV40GFP, which expresses GFP from the SV40 promoter (14, 55). TB40E-IE2YFP allows fluorescent detection of the virus only if the MIEP is active. SV40GFP allows fluorescent detection of all infected cells in which the SV40 promoter is active.

### Generation of YY1-edited iPSC and THP1 cells using CRISPR/Cas9.

Guide RNA to YY1 was delivered to cells via lentivirus, which was generated by cloning the sequence GATGTAGAGGGTGTCGCCCG into the lentiGuide-Puro plasmid (Addgene; number 52963), according to the protocol described by Shalem et al. (56). Alongside the lentiviral packaging vector, psPax, and the envelope vector, pMD.2G, this was transfected into HEK293T cells using Lipofectamine according to the manufacturer’s instructions. At 48 h posttransfection, lentivirus in the supernatant was harvested, passed through a 0.45-μm syringe filter, and used to transduce THP1 cells and iPSCs stably expressing Cas9. Cells were then selected with puromycin until all control cells had died. THP1 cells and iPSCs stably expressing Cas9 had been generated using lentivirus derived from lentiCas9-BLAST (Addgene; number 52962).

### Phenotyping of WT THP1 cells and YY1-KO THP1 cells.

WT THP1 cells and YY1-KO THP1 cells treated with PMA (Sigma) to differentiate them (or left untreated) were harvested and washed in phosphate-buffered saline without calcium or magnesium (DPBS) (Sigma). The cells were then blocked with 1/50 normal mouse serum (Invitrogen) and TruStain FcX (BioLegend) at the manufacturers’ recommended levels for 10 min at room temperature. Each sample was then split and stained with the following mixture: True-Stain Monocyte Blocker (BioLegend), LIVE/DEAD aqua (Invitrogen), and HLA-DR brilliant violet (BV) 421 (clone L243), CD163 BV 605 (clone GHI/61), CD16 BV 650 (clone 3G8), CD14 peridinin chlorophyll protein (PerCP)-Cy5.5 (clone M5E2), CD206 phycoerythrin (PE) (clone 15-2), CD64 PE Dazzle 594 (PE-Dz 594) (clone 10.1), CD80 PE-Cy5 (clone 2D10), CD86 PE-Cy7 (clone IT2.2), CD209 allophycocyanin (APC) (clone 9E9A8), HLA ABC Alexa Fluor (AxF) 700 (clone W6/32), and CD68 APC-Cy7 (clone Y1/82A) (all BioLegend) or True-Stain monocyte blocker, LIVE/DEAD Aqua, IgG2a BV 421, IgG1 BV 605, IgG1 BV 650, IgG2a PerCP-Cy5.5, IgG1 PE, IgG1 PE-Dz 594, IgG1 PE-Cy5, IgG2b PE-Cy7, IgG2a APC, IgG2a AxF 700, and IgG2b APC-Cy7 (all isotypes BioLegend), for 30 min at 4°C. Cells were washed in excess DPBS and then fixed with a 2% paraformaldehyde (PFA) solution. Samples were kept in the dark at 4°C until acquisition on an LSR Fortessa (BD Biosciences) using FACS Diva software. Samples were then analyzed using FlowJo, by using first a time-versus-side scatter gate, to identify the main flow of cells, and then these cells were gated for single cells (forward scatter area versus forward scatter height), live cells (forward scatter width versus LIVE/DEAD aqua dye), and then THP1 cells (forward scatter area versus side scatter area [log scale]). Phenotype markers were analyzed from this gate. The gating strategy for this phenotype panel was optimized using fluorescence-minus-one controls and matching isotype control staining for each cell type.

### TGFbeta ELISA.

The human TGFbeta1 ELISA kit (Invitrogen) was used for analysis of levels of secreted TGFbeta. This assay includes an acidification step which allows total TGFbeta (both latent and active forms) to be assayed.

### Chromatin immunoprecipitation assays.

Chromatin immunoprecipitations (ChIPs) were carried out using the Imprint ChIP kit (Sigma) using the manufacturer protocol.

### Drugs and treatments.

Recombinant TGF (R&D) was resuspended in phosphate-buffered saline (PBS) and added to cells at the desired concentrations. LDN 193189 (LDN; Sigma), SB431542 (Sigma), and SD208 (Sigma) were reconstituted using the company directions and at the desired concentrations. These recommended concentrations of the drugs were found not to be toxic to the cells as determined by trypan blue exclusion.

### Western blotting.

Cell lysates were analyzed by Western blotting using the following primary antibodies: SMAD2 (Cell Signaling) SMAD2-P (Ser465 and Ser467; Thermo Fisher), YY1 (Santa Cruz), SMAD6 (C terminal; Sigma), and actin (Abcam), followed by the appropriate horseradish peroxidase (HRP)-conjugated secondary antibody, and analyzed by chemiluminescence.

### Immunofluorescence.

Adherent cells were fixed with 4% paraformaldehyde for 20 min before permeabilization with 0.5% Triton X-100 for 20 min and blocking in 3% bovine serum albumin (BSA)/PBS for 1 h. After this time, primary antibodies were added. Differentiation markers Oct4 (Stemgent), Nanog (Stemgent), and repressive transcription factor YY1 (Santa Cruz) antibodies were all added at 1:200 for 1 h and after washing with the relevant Alexa Fluor 588 secondary antibody with Hoechst for 1 h before visualization by fluorescence microscopy.

### RT-qPCR.

RT-qPCR analyses were carried out using the QuantiTect (Qiagen) SYBR kit using standard primers and parameters for glyceraldehyde-3-phosphate dehydrogenase (GAPDH) and specific IE primers previously described (57). SMAD6 and YY1 primers were obtained from Santa Cruz and used at the defined manufacturer’s parameters.

### qPCR.

The MIEP was detected with previously designed primers (57) and analyzed using qPCR with SYBR green (Qiagen) using standard parameters. Alternatively, for viral genome quantification, gB primers (Gensig) were used alongside GAPDH primers (Qiagen) and the virus QuantiTect SYBR kit (Qiagen) was used for analysis as described previously (57).

### miRNA and siRNA analyses.

Total miRNA was isolated from cells using the miRNeasy kit from Qiagen. RNA was then analyzed in a 2-step SYBR RT-PCR using the hsa-miR-29a or hsa-miR-16 kit (Generon). Antagomir to hsa-miR-92a was obtained from Sigma. siRNA to SMAD6 or scramble siRNA was obtained from Sigma and electroporated by nucleofection using standard THP1 cell parameters (Nucleofector). Delta delta threshold cycle (*C_T_*) values were determined relative to the housekeeping hsa-miR-16, as previously published (54).

### T cell killing assay.

T cell killing assays were carried out as described previously (8, 18, 55, 58).
