# Supplementary figures and images for: A BMPR2/YY1 Signaling Axis Is Required for Human Cytomegalovirus Latency in Undifferentiated Myeloid Cells
Source: mBio. 2021 Jun 1;12(3):e00227-21. doi: 10.1128/mBio.00227-21 (PMC8262994; doi:10.1128/mBio.00227-21)

S1A

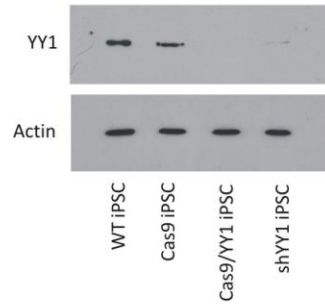

S1B

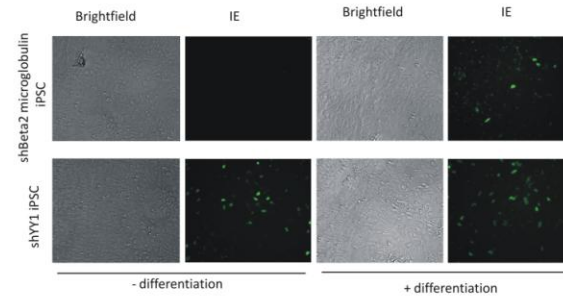

S1C

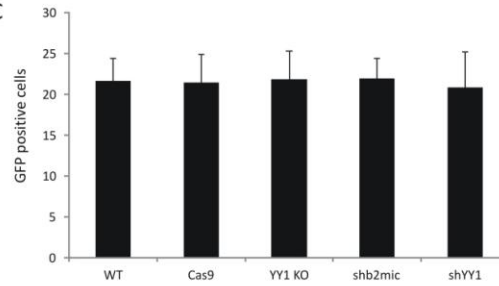

S1D

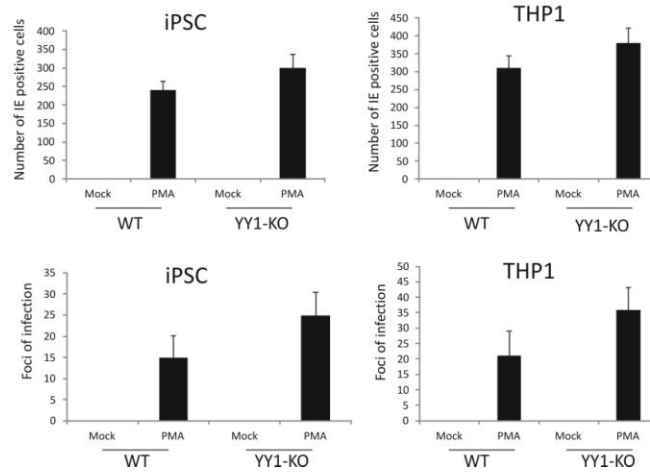

Supplement: FIG S1 [file mbio.00227-21-sf001.pdf]
